# Identification and prognostic prediction of high-risk multiple myeloma by exosomal microRNA

**DOI:** 10.3389/fonc.2025.1659708

**Published:** 2025-10-31

**Authors:** Ying Tian, Wenjiao Tang, Chunlan Zhang, Juan Xu, Yunfan Yang, Qinyu Liu, Xushu Zhong, Jie Wang, Caigang Xu

**Affiliations:** ^1^ Department of Hematology, West China Hospital, Sichuan University, Chengdu, Sichuan, China; ^2^ Chengdu Shang Jin Nan Fu Hospital/Shang Jin Hospital of West China Hospital, Sichuan University, Chengdu, Sichuan, China

**Keywords:** exosomal miRNA, multiple myeloma, diagnostic, prognostic, liquid biopsy

## Abstract

**Introduction:**

Circulating exosomal miRNAs have emerged as important tools for liquid biopsy in cancer diagnosis and prognosis prediction. This project establishes a plasma exosomal miRNA-based system for diagnosing multiple myeloma(MM) and evaluating patient prognosis, aiming to provide novel strategies for diagnosis and early warning in high-risk MM patients.

**Methods:**

The study prospectively collected plasma samples and clinical data of newly diagnosed multiple myeloma (NDMM) patients and healthy controls (HCs). Plasma samples were obtained in MM patients before the administration of any chemotherapy. The study comprises three stages to identify plasma exosomal miRNAs associated with the diagnosis and prognosis of MM. In the screening stage, next-generation sequencing of plasma circulating exosomes was performed in 17 NDMM patients and 8 HCs to screen the candidate differentially expressed miRNAs. We further investigated a testing stage of 80 individuals (including 60 NDMM patients and 20 HCs) and a verification stage of 130 NDMM through qPT-PCR.

**Results:**

Utilizing a testing cohort of 60 newly diagnosed MM cases, we developed a diagnostic model based on six miRNAs (hsa-miR-192-5p, hsa-miR-10a-5p, hsa-miR-10b-3p, hsa-miR-148a-3p, hsa-miR-193b-5p, hsa-miR-483-3p) achieving an AUC of 0.94, sensitivity of 0.88, and specificity of 0.94. In a validation cohort of 130 MM patients, we developed a prognostic nomogram that amalgamated the expression levels of three key exosomal miRNAs (hsa-miR-193b-5p, miR-483-3p, and let-7b-5p) with critical clinical variables, which exhibits superior performance compared to the ISS staging system. This integrative model effectively predicted 1-, 3-, and 5-year survival probabilities, thereby stratifying patients into distinct risk categories for enhanced clinical decision-making and personalized follow-up strategies.

**Discussion:**

This research validates the diagnostic and prognostic utility of exosomal miRNA models in MM, emphasizing their discriminative and predictive capabilities.

## 1 Introduction

Multiple myeloma (MM) is the second most common hematological malignancy characterized by anemia, renal insufficiency, hypercalcemia and bone disease. This disease is still incurable, especially for high-risk patients (1, 2). Early identification of high-risk patients and timely active treatment are critical to improving the overall survival of MM patients (3–5). However, MM is a highly heterogeneous disease, and there is still a lack of a unified understanding of the definition of high-risk MM (6, 7). Despite the identification of various biomarkers and staging systems to categorize the prognosis of MM, significant disparities in survival rates persist among patients classified within the same stage. For instance, the extensively utilized R-ISS staging system is capable of discerning the prognosis for only a minority of stage III patients, while those in stage II exhibit a spectrum of heterogeneity, leading to diverse survival outcomes (8, 9). The existing staging paradigm is heavily predicated on the cytogenetic features of bone marrow. However, a single-site bone marrow aspiration may fail to precisely gauge the tumor burden and biological profile in certain instances of heterogeneous MM characterized by localized tumor bone marrow infiltration (10, 11). Furthermore, bone marrow aspiration, being an invasive procedure, is not only challenging for some elderly and frail patients to endure but also occasionally unattainable in certain cases. Consequently, there is a pressing need to refine the current prognostic staging systems and to identify less invasive biomarkers that can reliably forecast patient outcomes. In this context, liquid biopsy has garnered considerable interest, with exosomes emerging as a promising candidate for non-invasive cancer detection and prognosis (12–14).

Exosomes, nanoscale vesicles measuring 30–150 nm and derived from cells, are enveloped by a phospholipid membrane and are rich in proteins, lipids, and nucleic acids. They facilitate intercellular communication by transferring genetic and protein information, playing roles in inflammation, tissue repair, and cancer spread (15). The protective membrane of exosomes shields their RNA content from degradation, making it more stable and concentrated than somatic RNAs (16, 17). Advances in RNA sequencing have revealed that numerous microRNAs (miRNAs) are instrumental in various cancers and can modulate the tumor microenvironment and immune signaling via exosomes (18, 19). Exosomal miRNAs have been shown to impact tumor growth, metastasis, and the tumor microenvironment by transmitting signals between cells and even entering the bloodstream for long-distance communication (20, 21).

Studies have identified circulating exosomal miRNAs as potential biomarkers for early cancer detection, treatment monitoring, and prognosis (22, 23). Despite the potential of exosomes as liquid biopsy biomarkers, the role and prognostic significance of miRNAs in MM exosomes remain understudied. Further research is needed to clarify their potential as prognostic indicators in MM.

This study aims to establish a diagnostic and prognostic evaluation system for MM based on plasma exosomal miRNA, providing new strategies for diagnosis and early prediction of high-risk MM.

## 2 Patients and methods

**Figure 1:**
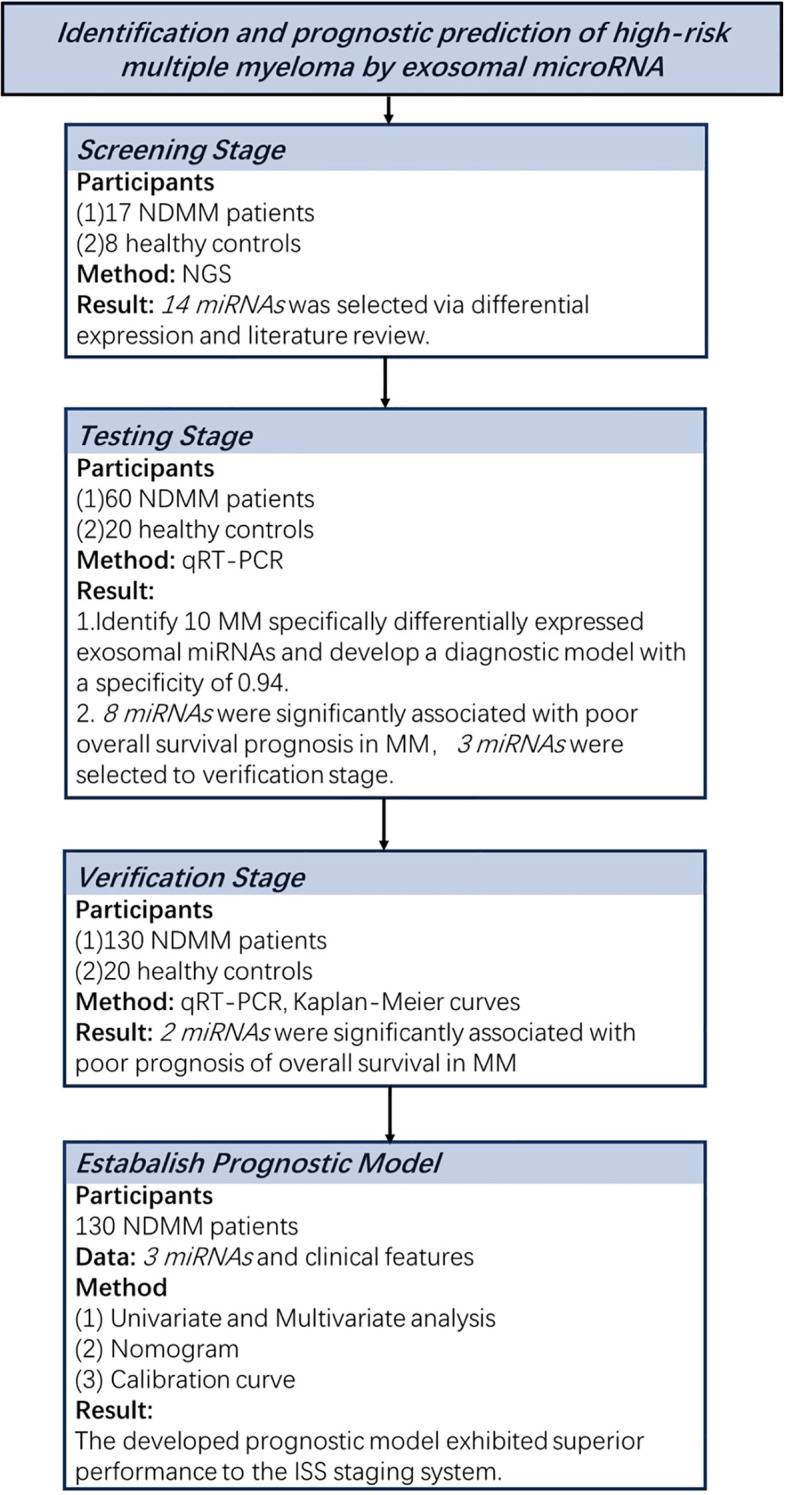
Overview of the strategy for investigating exosomal miRNAs and developing diagnostic and prognostic models in multiple myeloma.

**Figure 1** shows the flow chart of the study.

### 2.1 Patients

The study prospectively collected plasma samples and clinical data of newly diagnosed multiple myeloma (NDMM) patients and healthy controls (HCs) at West China Hospital, Sichuan University from July 1, 2012, to January 30, 2018 and the follow-up period ended on July 12, 2024. Plasma samples were obtained in MM patients before the administration of any chemotherapy. The health status of control individuals was confirmed by routine physical examinations at the physical examinations center. This study was approved by the Institutional Review Board of West China Hospital. All participants provided written informed consent for blood collection and follow-up.

Myeloma diagnosis was strictly based on the diagnostic guidelines of the International Myeloma Working Group (IMWG). The study comprises three stages to identify plasma exosomal miRNAs associated with the diagnosis and prognosis of MM. In the screening stage, next-generation sequencing of plasma circulating exosomes was performed in 17 NDMM patients and 8 HCs to screen the candidate differentially expressed miRNAs. We further investigated a testing stage of 80 individuals (including 60 NDMM patients and 20 HCs) and a verification stage of 130 NDMM through qPT-PCR.

### 2.2 Exosome isolation and identification

Peripheral blood samples from individuals were collected in EDTA tubes following a regular venipuncture procedure. After centrifugation at 3,000×g for 15 min at 4°C, the plasma was aspirated and stored at −80°C before use. The exosomes were isolated using the size exclusion chromatography (SEC) methods described previously, with minor modifications (24). Briefly, 1 mL of 0.8μm-filtered blood plasma was 1.5-fold diluted with PBS and further purified using Exosupur^®^ columns (Echobiotech, China). The samples were then eluted with a further 0.1 M PBS and 2 mL eluate fractions were collected according to the manufacturer’s instructions. Fractions were concentrated to 200μL by 100kDa molecular weight cut-off Amicon^®^ Ultra spin filters (Merck, Germany).

The identification of exosomes was performed by three methods including nanoparticle tracking analysis (NTA), transmission electron microscopy (TEM) and western blot analysis using rabbit polyclonal antibody CD9 (60232-I-Ig, Proteintech, Rosemont, IL), Alix (sc-53540, Santa Cruz, CA, USA), TSG101 (sc-13611, Santa Cruz, CA, USA) and calnexin (10427-2-AP, Promega, Madison, WI). The details of the identification methods were described in the **Supplementary Figure S1**.

### 2.3 Exsomal RNA isolation and RNA analyses

Total RNA was extracted and purified from plasma exosome using miRNeasy Plasma Advanced Kit (Qiagen, cat. No. 217204) according to the kit instruction. RNA concentration and purity were evaluated using the RNA Nano 6000 Assay Kit of the Agilent Bioanalyzer 2100 System (Agilent Technologies, CA, USA).

### 2.4 Next-generation sequencing of exosomal RNAs

In the case of small RNA libraries, an input material 3 ng of RNA per sample was used and sequencing libraries were generated using the QIAseq miRNA Library Kit (Qiagen, Frederick, MD) following the provided recommendations, and index codes were added for sample traceability. Reverse transcription (RT) primers with unique molecular indices (UMIs) were introduced to analyze the quantification of miRNA expressions during cDNA synthesis and PCR amplification. The quality of the libraries was meticulously evaluated using the Agilent Bioanalyzer 2100 and qPCR. Subsequently, the index-coded samples were clustered on the cBot Cluster Generation System with the TruSeq PE Cluster Kit v3-cBot-HS from Illumina (Illumina, San Diego, CA, USA) strictly adhering to the manufacturer’s protocol. Following the cluster generation, the library preparations underwent sequencing on the Illumina Novaseq 6000 platform, yielding paired-end reads at EchoBiotech Co. Ltd., Beijing, P. R. China.

### 2.5 Identification and analysis of differentially expressed miRNAs between multiple myeloma and healthy controls

We meticulously cleaned the reads and aligned them against the Silva database, GtRNAdb database, Rfam database, and Repbase database to effectively filter out ribosomal RNA (rRNA), transfer RNA (tRNA), small nuclear RNA (snRNA), small nucleolar RNA (snoRNA), and other non-coding RNAs (ncRNAs) as well as repetitive sequences by employing Bowtie tools with a soft alignment approach. The reads that remained after this rigorous filtering process were then utilized to detect both known and novel miRNAs. This was achieved by comparing them against the known miRNAs catalogued in miRbase and the Human Genome reference (GRCh38). For each miRNA identified, we derived a read count from the mapping results and subsequently calculated the Transcripts Per Million (TPM) to quantify their expression levels. Differential expression analysis of control vs MM was performed using the EdgeR package with cutoff P-value ≤ 0.05 and |log2Fold change|≥0.58.

We then harnessed the power of the multiMiR R package to conduct a thorough analysis of target genes associated with differentially expressed miRNAs. To ensure the reliability of our predictions, we implemented a stringent filtering criterion based on the consensus prediction across databases and experimental validation. Specifically, we selected miRNA-target gene pairs that were either predicted by at least three distinct databases/software tools or had been experimentally validated at least once. Subsequently, we performed a functional annotation and enrichment analysis to characterize the biological significance of the differentially expressed miRNA target genes. Gene Ontology (GO) enrichment analysis of the target genes of differentially expressed miRNAs was implemented by the topGO R packages. KOBAS software was used to test the statistical enrichment of differential expression genes in KEGG pathways and the “ggplot” software package was used to visualize the results.

### 2.6 Verification of differentially expressed exosomal miRNAs in multiple myeloma and healthy controls using qRT-PCR

Total RNA was extracted and purified from plasma sEVs using miRNeasy Serum/Plasma Advanced Kit (Qiagen, cat. No. 217204). TaqMan™ advanced miRNA assays were performed for miRNA quantification using PrimeScript™ RT reagent Kit (Perfect Real Time) (TAKARA, RR037A) and TaKaRa Ex Taq Hot Start Version (Takara, Carlsbad, CA, cat. No. RR006A). The relative expression level of the target miRNA was calculated using the 2^-ΔΔCt method. All experiments were set up with three replicate wells and repeated three times. Each biomarker’s CT value was averaged across triplicate wells per sample, and ΔCT was determined by subtracting the U6 internal control CT value from the biomarker’s CT value. The max ΔCT for each biomarker was used for normalization; -ΔΔCT=-(sample ΔCT - max ΔCT), followed by conversion to 2^(-ΔΔCT). The miRNA expression cut-off was calculated using ROC by classifying death within two years as positive.

### 2.7 Statistical analysis

Nonparametric Mann–Whitney or Kruskal–Wallis tests were used to compare relative miRNA expression levels between different groups; Kaplan–Meier method and log‐rank test were applied to plot overall survival (OS); Cox proportional hazard model was employed to identify independent outcome predictor and compute hazard ratio. Based on the results of multivariate analysis, nomograms for 1-year, 3-year and 5-year OS were constructed. The calibration curves were built to identify whether the predicted and actual survival were consistent. The receiver operating characteristic (ROC) curve and logistic regression were adopted to estimate the diagnostic value of the candidate miRNAs. The prognostic performance and discriminatory power of two models (ISS stage and norm-stage) were measured by AUC. For missing values, we use the multiple imputation method with random forest to generate five datasets and then take the average to obtain a new dataset for analysis. The data characteristics before and after imputation are shown in **Supplementary Table S1**. All statistical analyses were performed using R software (version 4.4.1). The R package included survival, survminer, rms, ggplot2, pROC, mice, forestmodel. For each analysis, P less than or equal to 0.05 was the threshold for significance.

## 3 Results

### 3.1 Characteristics of the enrolled participants

We prospectively enrolled 175 individuals from West China Hospital, Sichuan University, including 25 subjects (17 NDMM patients and 8 HCs) in the screening stage, 80 subjects (60 NDMM patients and 20 HCs) in the testing stage, 130 subjects (60 NDMM patients from testing stage and 70 other NDMM patients) in the verification stage. The characteristics of the 175 participants are presented in **Table 1**.

**Table 1:** Baseline characteristics of newly diagnosed multiple myeloma and healthy controls.

Variables	Screening stage		Validation stage	
	NDMM n=17	HC n=8	NDMM n=130	HC n=20
Gender				
Male	10 (58.8%)	4 (50%)	80 (61.5%)	10 (50%)
Female	7 (41.2%)	4 (50%)	50 (38.5%)	10 (50%)
Age (median, range)	61 (37-74)	63.5 (49-72)	62 (37-79)	63 (50.5-71)
ISS stage				
I	3 (17.6%)	/	26 (20%)	/
II	7 (41.2%)	/	46 (35.4%)	/
III	7 (41.2%)	/	58 (44.6%)	/
Lactate dehydrogenase (median, U/L)	209 (148.50,295)	/	164.5 (134,204.5)	/
Creatinine (median, mg/dl)	1.05 (0.85,2.07)	/	0.88 (0.73,1.34)	/
Serum Calcium (median, mmol/L)	2.33 (2.18,2.44)	/	2.24 (2.09,2.38)	/
White Blood Cell (median, 10^9/L)	5.06 (4.05,5.80)	/	5.32 (4,7.22)	/
Hemoglobin (median, g/L)	98 (74,118.50)	/	93 (73,114.25)	/
Platelets (median, 10^9/L)	138 (97,175.5)	/	156 (113.5,198)	/
Albumin (median, g/L)	37.20 (34.15,46.05)	/	35.8 (29.78,39.95)	/
Total Protein (median, g/L)	80.50 (69.45,104.40)	/	84.75 (70.95,106.7)	/
Globulin (median, g/L)	39.10 (27.85,69.25)	/	48.3 (30.23,73.85)	/
Alkaline phosphatase (median, U/L)	76 (55.50,106.5)		76 (56.75,104.25)	/
Monoclonal protein (median, g/L)	33.9 (18.55,41.53)	/	33.95 (13.03,48.03)	/
Bone marrow plasma cell ratio (median, %)	45 (11,65.5)	/	33.75 (16,51.75)	/
Immunoparesis degree*				
No	1 (5.88%)	/	11 (8.5%)	/
Mild	0 (0%)	/	12 (9.2%)	/
Moderate	5 (29.41%)	/	21 (16.2%)	/
Severe	11 (64.70%)	/	86 (66.2%)	/
Heavy-chain type				
No	6 (35.29%)	/	36 (27.7%)	/
IgG	6 (35.29%)	/	66 (50.8%)	/
IgA	5 (29.41%)	/	28 (21.5%)	/
Light-chain type				
kappa	8 (47.06%)	/	65 (50%)	/
lambda	9 (52.94%)	/	65 (50%)	/

NDMM, newly diagnosed multiple myeloma; HC, healthy controls.

*Immunoparesis Criteria: At least one unaffected immunoglobulin level is below the normal lower limit (IgG<7g/L, IgA<0.7g/L, or IgM<0.4g/L). The degree of immunoparesis is classified into four levels based on the level of unaffected immunoglobulin quantification below the normal lower limit: None (above the lower limit of normal, IgG ≥7g/L, IgA≥ 0.7g/L, IgM≥0.4g/L), Mild (0~25% below the normal lower limit), Moderate (25%~50% below the normal lower limit), Severe (>50% below the normal lower limit) (25, 26).

### 3.2 Exosome identification and circulating exosomal miRNA profiling

The morphology and size distribution of exosomes isolated from the plasma of both NDMM patients and healthy controls were subsequently characterized by transmission electron microscopy (TEM) and nanoparticle tracking analysis (NTA). The analysis revealed that the exosomes appeared as cup‐shaped vesicles between 80 and 95 nm in diameter (**Supplementary Figure S1**). Furthermore, the presence of exosome-specific markers, including CD9, TSG101, and Alix, was confirmed in the enriched fractions obtained from the plasma. In contrast, Calnexin, a protein that serves as a negative control for sEVs, was notably absent in exosomes (**Supplementary Figure S1**).

**Figure 2:**
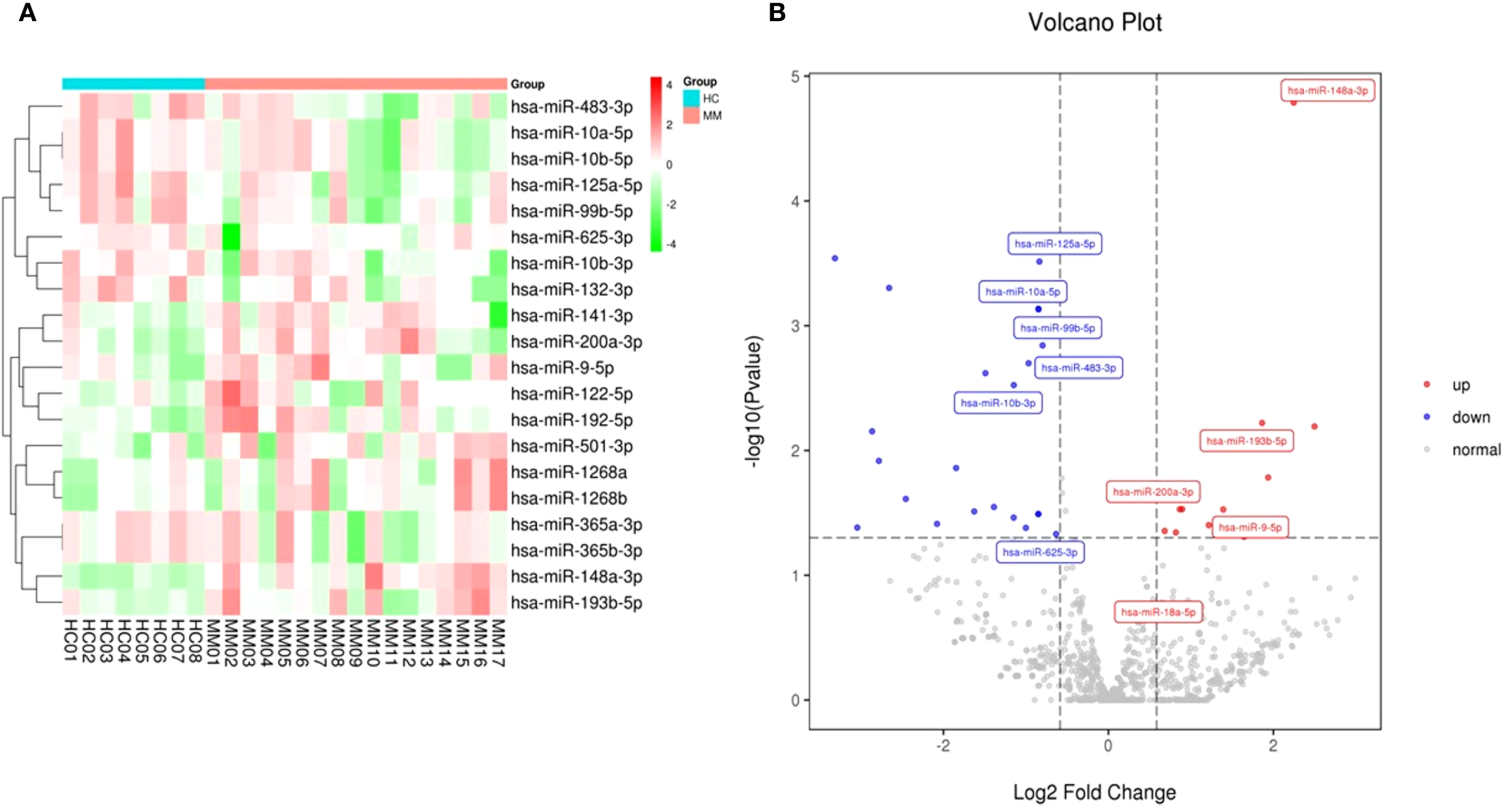
Clustering and volcano plots of differentially expressed miRNAs in circulating exosomes between NDMM and HCs. **(A)** The heat map shows the top 10 miRNAs with the most significant upregulation and downregulation; **(B)** Volcano plot depicting the differential expression of miRNAs in exosomes from MM patients versus healthy controls.

A total of 1,314 miRNAs including 1191 known miRNAs and 123 newly-predicted miRNAs were detected by next-generation sequencing. Compared to the healthy control group, we found that 10 miRNAs were significantly upregulated and 10 miRNAs were significantly downregulated. The clustering analysis and volcano plots of differentially expressed miRNAs are shown in **Figure 2**, in which miR-148a-3p was significantly highly expressed in MM compared to the healthy control group (log2FoldChange value 2.246, P-value 0.000016).

### 3.3 GO and KEGG enrichment analyses results

**Figure 3:**
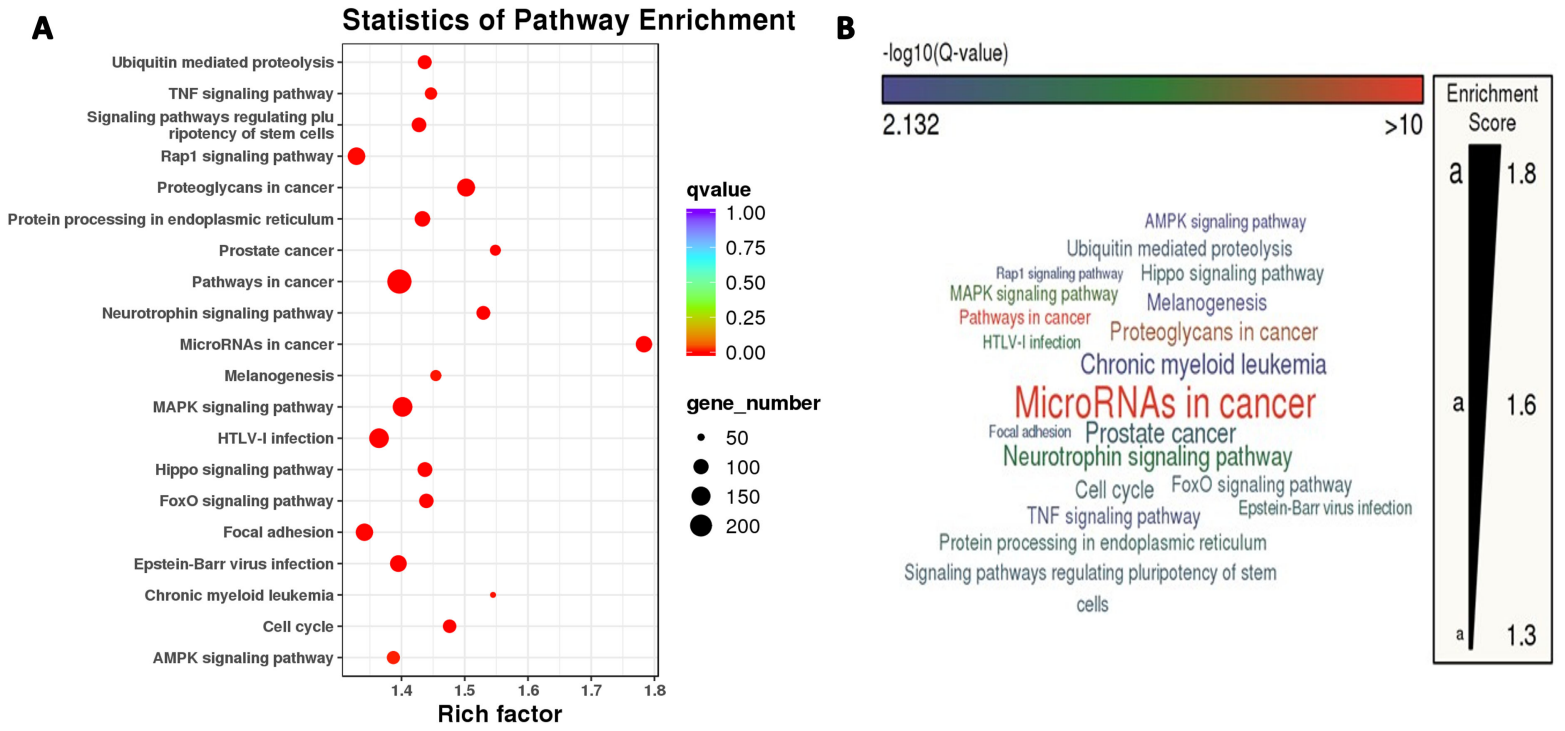
Two panels show pathway enrichment analysis. **(A)** This is a bubble chart with pathways on the y-axis and rich factor on the x-axis. Bubble size indicates gene number and color indicates q-value. **(B)** This is a word cloud of pathways, with size reflecting enrichment score.

GO entries enrichment containing biological process (BP), cellular component (CC), and molecular function (MF), with a P value screening for each component. In the up-regulation of miRNA target gene, the main results are the DNA damage response, signaling transduction by p53 class mediator resulting in cell cycle arrest, melanosome and double-stranded RNA binding (**Supplementary Figure S2**). Meanwhile, the core targets were subjected to the KEGG pathway enrichment analysis to screen for pathways with q<0.05, and a total of twenty were obtained, such as the MicroRNAs in cancer, the Thyroid hormone signaling pathway, the Neurotrophin signaling pathway, the Hippo signaling pathway, the FoxO signaling pathway, ErbB signaling pathway (**Supplementary Figure S2**).In the down-regulation of miRNA target gene, the main results are the regulation of RNA splicing, cytoplasmic stress granule, and monooxygenase activity (**Supplementary Figure S3**). Meanwhile, the core targets were subjected to the KEGG pathway enrichment analysis to screen for pathways with q<0.05, and a total of twenty were obtained, such as the MicroRNAs in cancer, the Neurotrophin signaling pathway, the mTOR signaling pathway, the TNF signaling pathway, the Rap1 signaling pathway, and the MAPK signaling pathway, the Insulin signaling pathway, the Hippo signaling pathway, the FoxO signaling pathway (**Supplementary Figure S3**). **Figure 3** illustrates the KEGG pathway enrichment analysis for differentially expressed microRNAs, with a primary focus on the “MicroRNAs in Cancer” pathway.

### 3.4 Identification of multiple myeloma specifically differentially expressed exosomal miRNAs

**Figure 4:**
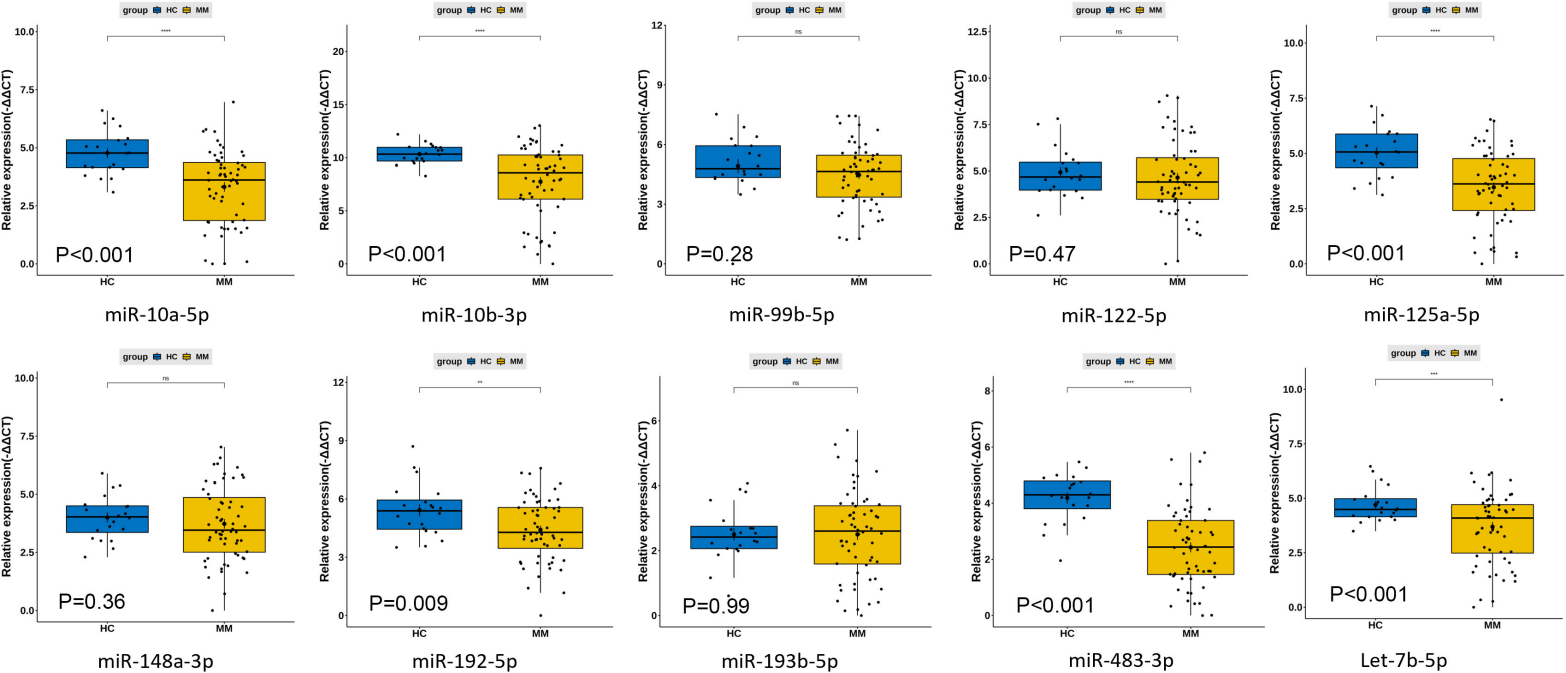
Differences in plasma circulating exosome miRNA qPCR expression levels between 60 newly diagnosed multiple myeloma patients and 20 healthy controls. **: p<0.05; ***: p<0.001; ns, not significant.

Based on the analysis of differentially expressed miRNAs between NDMM and HC and literature reviews (27), we selected fourteen miRNAs that met the criteria for fold change and statistical significance to underwent qRT-PCR in the testing stage including 60 NDMM patients and 20 HCs. The design of the primers of these fourteen miRNAs were described in the **Supplementary Table 2**. However, the presence of many unavailable values in the raw PCR data for hsa-miR-9-5p, hsa-miR-200a-3p, hsa-miR-625-3p, and hsa-miR-18a-5p, it is not possible to proceed with subsequent analysis. According to the expression levels of ten miRNAs in NDMM patients and HCs (**Supplementary Table 3**), six individual miRNAs (miR-483-3p, miR-10b-3p, miR-10a-5p, miR-125a-5p, let-7b-5p, miR-192-5p) were significantly decreased in MM (P<0.01) (**Figure 4**).

**Figure 5:**
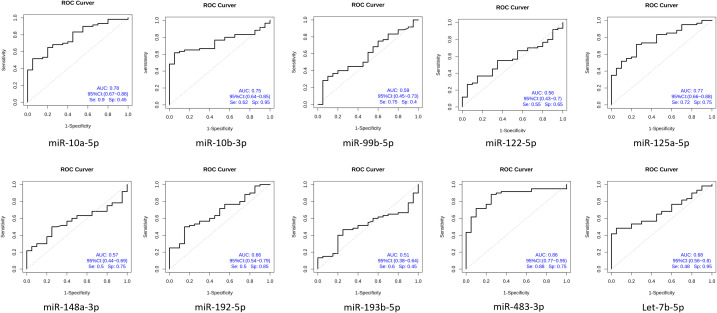
Circulating exosomal miRNA diagnostic test for newly diagnosed multiple myeloma patients.

**Figure 6:**
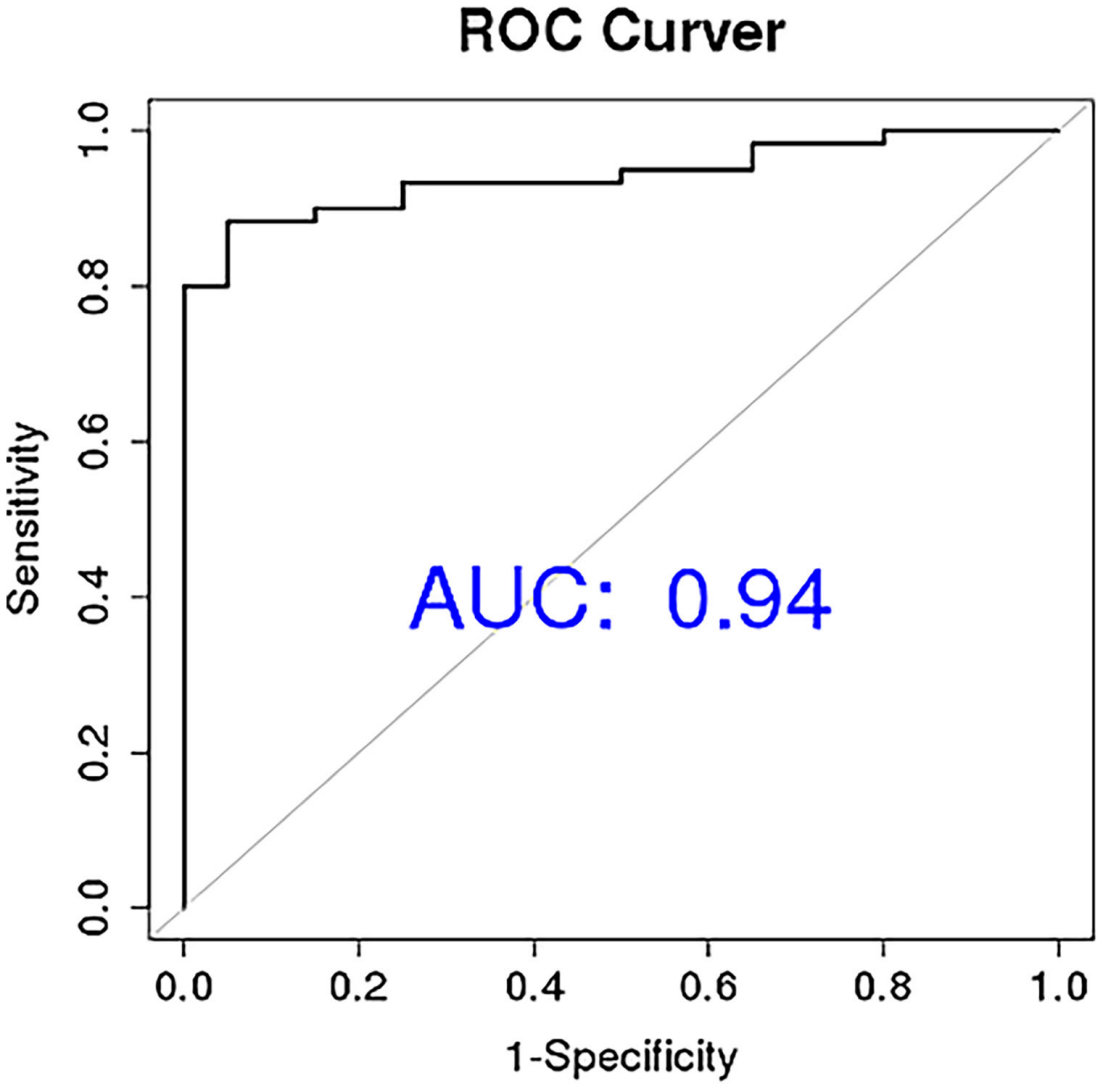
Circular extracellular vesicle miRNA combination diagnosis of newly diagnosed multiple myeloma patients ROC curve.

To determine the diagnostic performance characteristics of the ten miRNAs in distinguishing NDMM patients from HCs, we plotted ROC curves for each miRNA. The results show that the areas under the ROC curve (AUC) for miR-483-3p, hsa-miR-125a-5p, and hsa-miR-10b-3p in the diagnostic test are 0.86, 0.78, and 0.77 respectively. This suggests that the downregulation of plasma circulating exosomal miR-483-3p, hsa-miR-125a-5p, and hsa-miR-10b-3p has a high diagnostic value for MM (**Figure 5**). Multivariate logistic analysis revealed that the combination of hsa-miR-192-5p, hsa-miR-10a-5p, hsa-miR-10b-3p, hsa-miR-148a-3p, hsa-miR-193b-5p, and hsa-miR-483-3p has an AUC of 0.94, a sensitivity of 0.88, and a specificity of 0.94 for diagnosing MM (**Figure 6**).

### 3.5 Impact of circulating exosomal miRNAs on survival in patients with NDMMs

**Figure 7:**
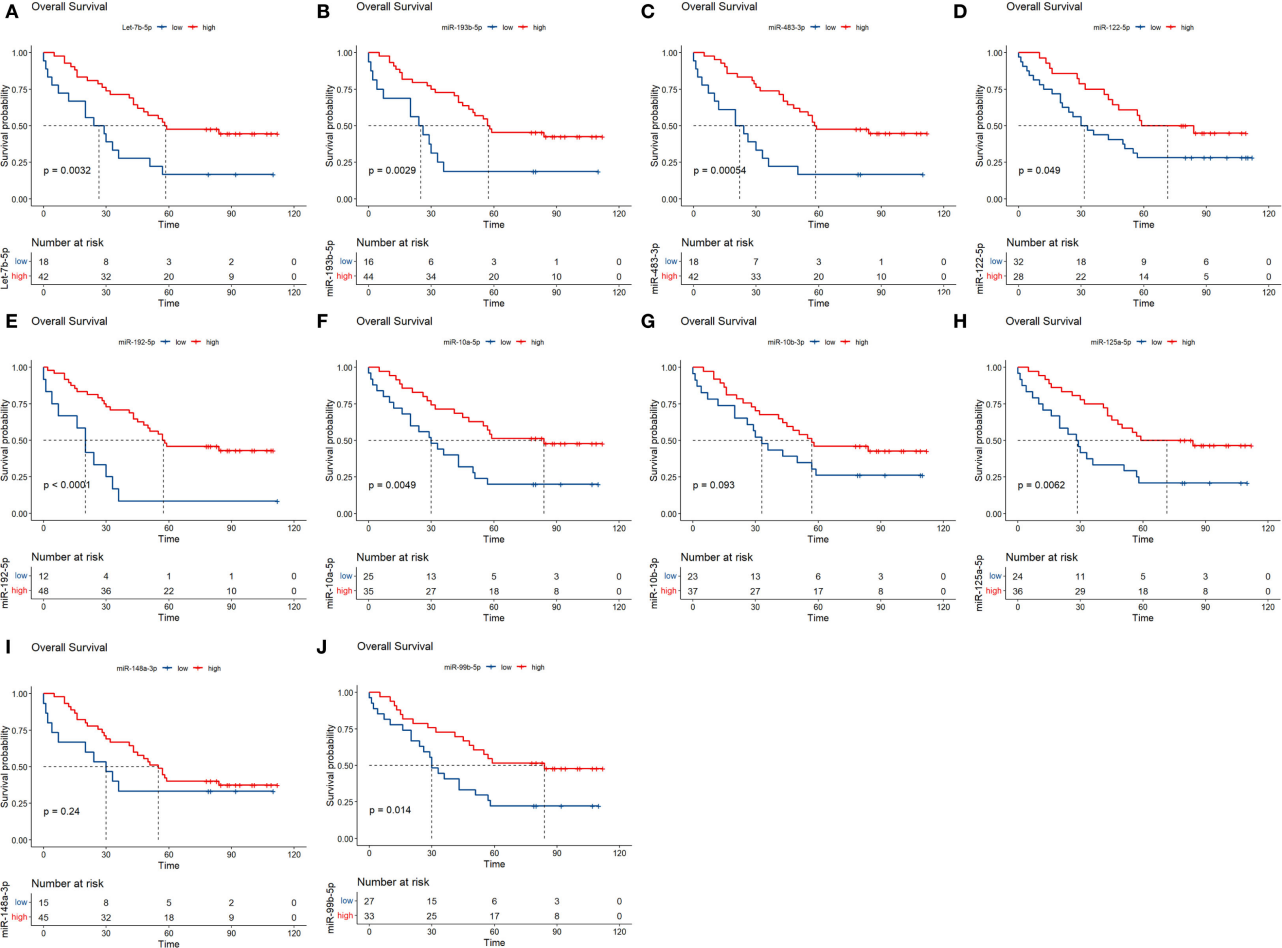
Analysis of prognostic markers of circulating exosomes in the overall survival of 60 newly diagnosed multiple myeloma in the testing set. **(A–J)** Each graph shows different microRNA. The x-axis represents time, and the y-axis represents survival probability. P-values are indicated, showing statistical significance. Red lines represent high expression while blue lines represent low expression. The number at risk is detailed below each graph.

The survival of 60 NDMM patients in the testing set were further analysed. The results showed that low expression of MM plasma circulating exosomal hsa-miR-193-5p, hsa-miR-483-3p, let-7b-5p, hsa-miR-122-5p, hsa-miR-192-5p, hsa-miR-10a-5p, hsa-miR-125a-5p and hsa-miR-99b-5p was significantly associated with poor overall survival prognosis in MM (**Figure 7**). Moreover, based on the P-values from the K-M analysis of 60 NDMM patients and previous literature reports (27), the prognostic value of hsa-miR-483-3p, let-7b-5p, and hsa-miR-193-5p was further validated in a larger cohort of 130 NDMM patients.

**Figure 8:**
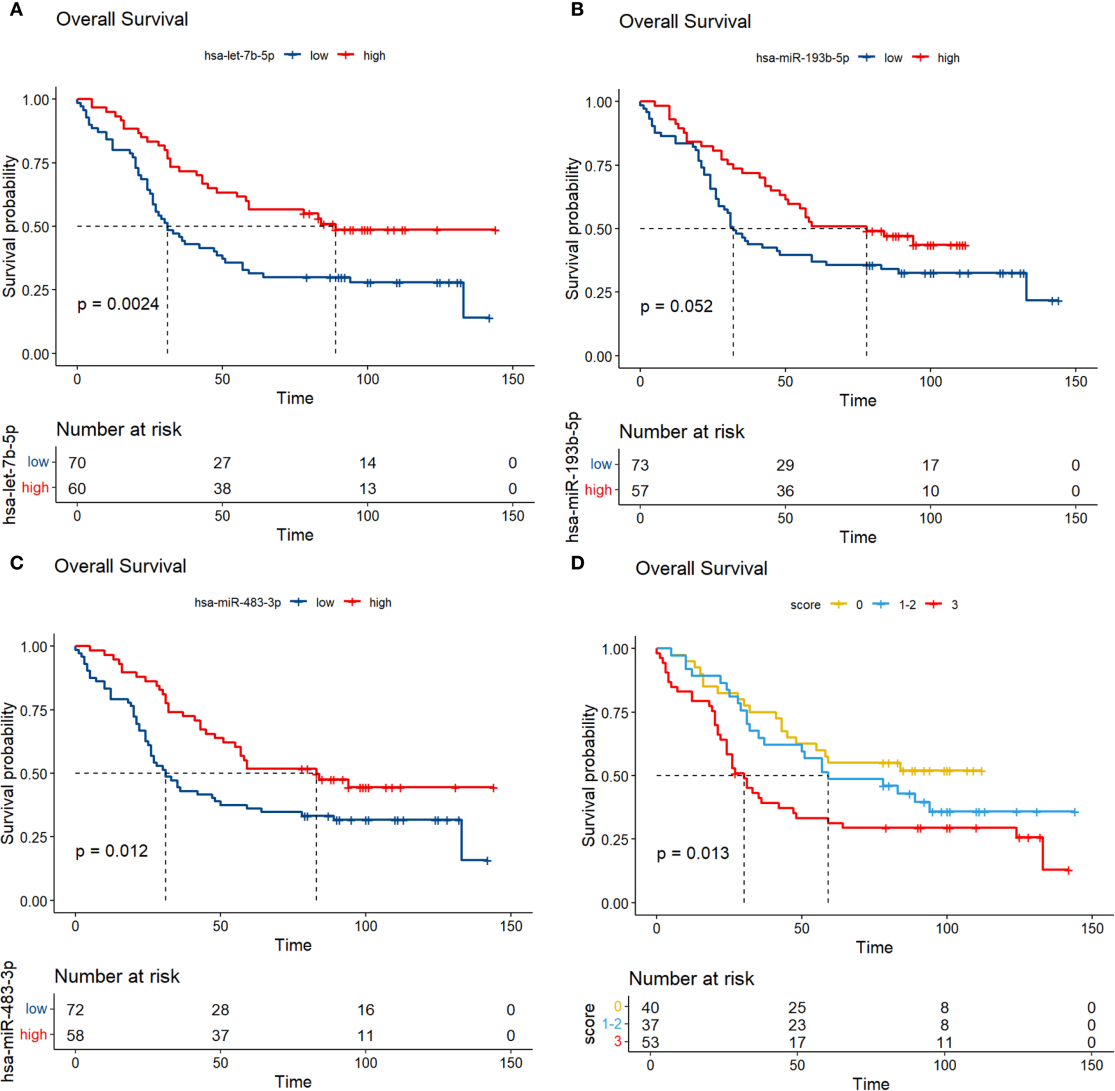
Analysis of prognostic markers of circulating exosomes in the overall survival of 130 newly diagnosed multiple myeloma in the verification set **(A-C)**; **(D)** K-M curves were plotted based on different scores in a validation set of 130 newly diagnosed multiple myeloma, with each low expression of the three prognostic biomarkers assigned a score of 1.

The results showed that low expression of hsa-miR-483-3p and let-7b-5p were significantly associated with poor prognosis of overall survival in MM, hsa-miR-193-5p showed no significant correlation with prognosis in the verification set (**Figure 8**). Previous studies identified miR-193b-3p as a top deregulated miRNA in myeloma cells and a direct regulator of the MCL1 transcript (28). Given this, and the borderline significant K-M analysis result (P < 0.1) observed for miR-193b-3p in our 130 NDMM patients, all three miRNAs (hsa-miR-483-3p, let-7b-5p, and hsa-miR-193-5p) were thus included in the subsequent analysis. We assigned a score of 1 to individuals with low miRNA expression and 0 to those with high expression. By integrating the expression profiles of has-miR-193b-5p, miR-483-3p and let-7b-5p, we constructed a novel variable, miRNA score, with a range from 0 to 3.

### 3.6 Prognostic prediction model for overall survival of NDMMs

**Figure 9:**
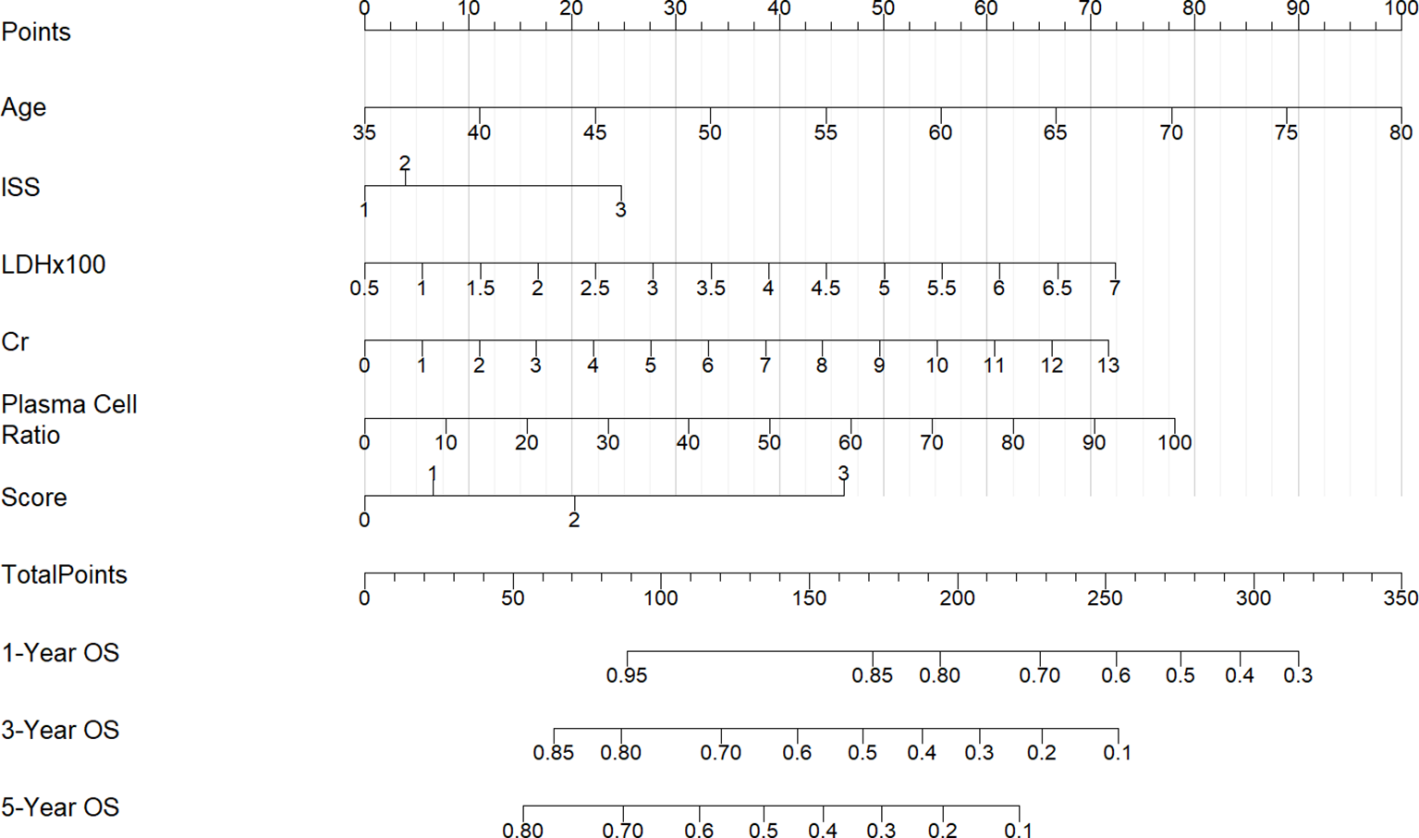
Nomograms for predicting the1-year, 3-year and 5-year overall survival rates of patients with NDMM.

**Figure 10:**
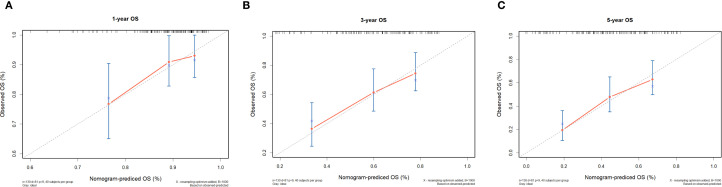
Calibration curves of the nomogram for 1-, 3-, and 5-year OS of NDMM patients. **(A)** shows one-year OS, **(B)** shows three-year OS, and **(C)** shows five-year OS. Each plot reflects increasing OS alignment over the years, highlighting the model's predictive accuracy over time.

In the 130 NDMM corhorts, univariate Cox regression analysis was performed to identify factors influencing OS and significant factors derived in the univariate Cox analysis were included in the multivariable analysis to identify independent factors correlated with prognosis in OS (**Table 2**). The multivariable analysis indicated that age, ISS stage, bone marrow plasma cell ratio and the previously identified differentially expressed miRNA combinations score were independently predictive of OS. Nomograms for predicting 1-year, 3-year and 5-year overall survival rates were established based on the results of multivariate Cox analysis (**Figure 9**). The calibration curves presented excellent coherence between the predictions and actual observations (**Figure 10**).

**Table 2:** Univariate and multivariable Cox regression analysis for overall survival of newly diagnosed multiple myeloma patients.

	Univariate Cox regression analysis			Multivariable Cox regression analysis		
Variables	HR	95% CI	*P* value	HR	95%CI	*P* value
Gender			0.12			
Male	ref					
Female	0.69	(0.43,1.10)				
Age	1.03	(1.00,1.05)	0.006	1.03	(1.01,1.06)	0.006
ISS stage			0.005			
I	ref			ref		
II	1.53	(0.76,3.10)	0.236	1.06	(0.5,2.23)	0.886
III	2.59	(1.34,5.03)	0.005	1.41	(0.67,2.99)	0.369
Lactate dehydrogenase	1.01	(1.00,1.01)	0.005	1.0	(1.00,1.00)	0.172
Creatinine	1.13	(1.04,1.23)	0.004	1.08	(0.97,1.20)	0.147
Serum Calcium	1.49	(0.84,2.64)	0.177			
Hemoglobin	0.99	(0.98,1.00)	0.072			
Monoclonal protein	0.99	(0.98,1.00)	0.156			
Bone marrow plasma cell ratio	1.01	(1.00,1.02)	0.007	1.01	(1.00,1.02)	0.029
Heavy-chain type			0.09			
No	ref					
IgG	0.60	(0.36,1.00)	0.051			
IgA	0.94	(0.52,1.70)	0.843			
Light-chain type						
kappa	ref					
lambda	1.31	(0.84,2.02)	0.232			
Immunoparesis degree			0.1			
No	ref					
Mild	1.44	(0.50,4.14)	0.503			
Moderate	0.65	(0.23,1.88)	0.431			
Severe	1.47	(0.63,3.40)	0.369			
miRNA score			0.04			
0	ref			ref		
1	1.280	(0.62,2.64)	0.504	1.10	(0.52,2.30)	0.808
2	1.406	(0.67,2.96)	0.369	1.33	(0.62,2.81)	0.463
3	2.171	(1.25,3.78)	0.006	1.90	(1.07,3.40)	0.029

### 3.7 Performance of the nomogram in stratifying risk

**Figure 11:**
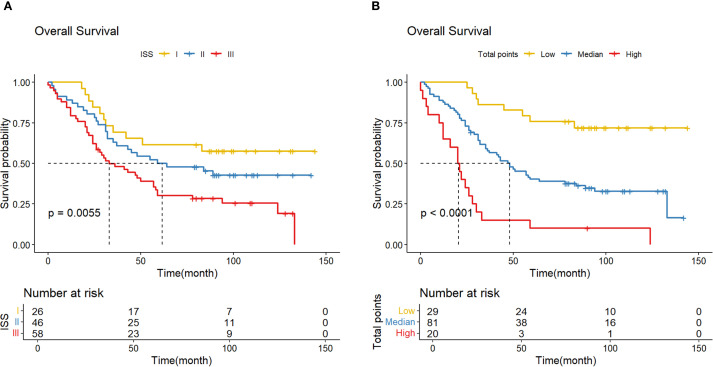
**(A)** K-M curve shows the difference in OS due to the ISS stage; **(B)** K-M curve shows the difference in OS due to the norm- stage.

**Figure 12:**
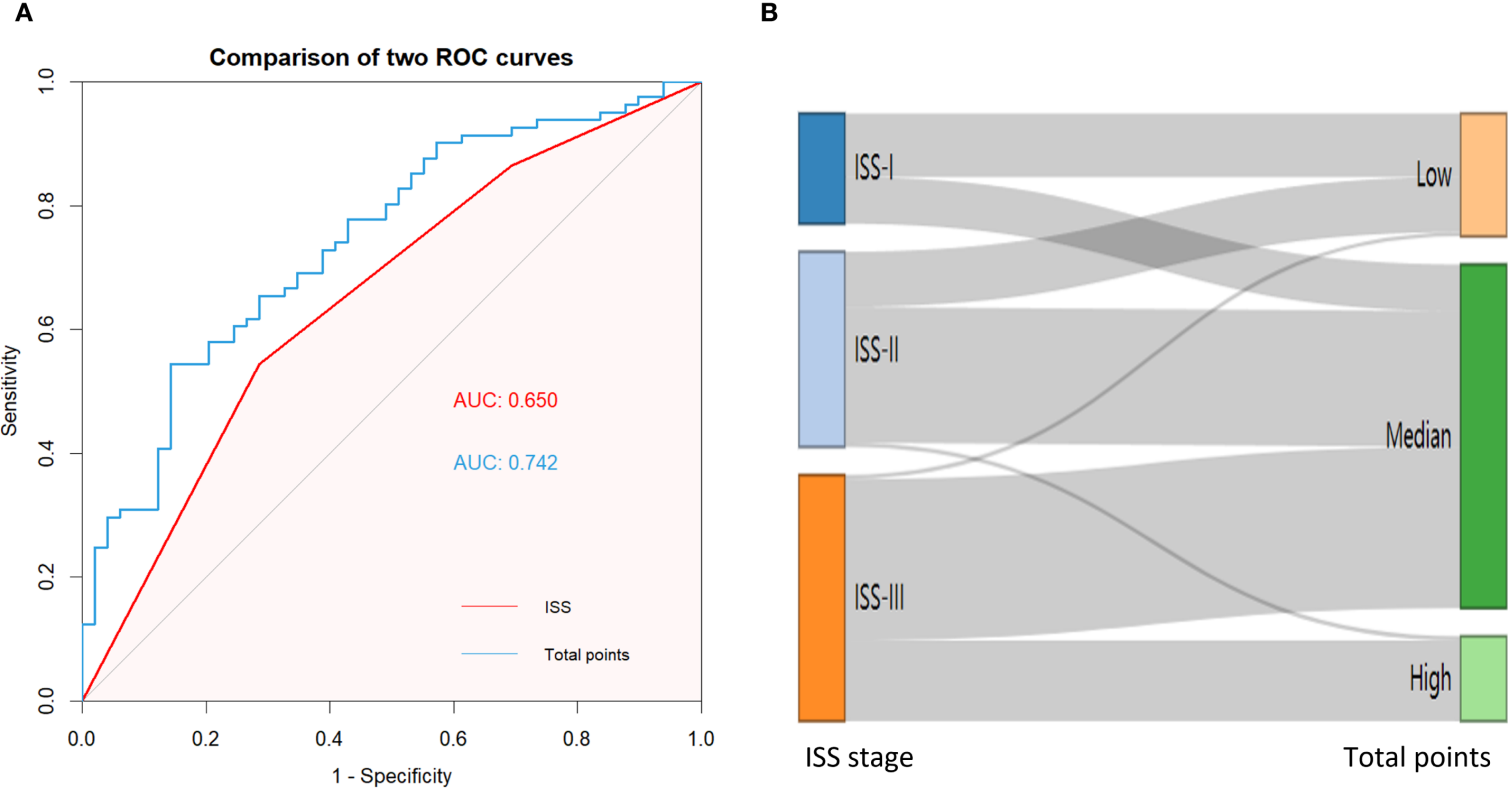
**(A)** AUC curve of the ISS and norm- stage for predicting OS; **(B)** An alluvial plot shows the association of norm- stage with ISS stage.

To further verify the feasibility of our prediction model, patients in the validation cohort were stratified into low-, median- and high-risk groups according to the scores generated by the OS nomogram. The cut-off values were 88.30-220.91 as determined by X-tile software. The Kaplan-Meier survival curves showed that high-risk patients had the worst OS, and the low-risk patients had the best OS (p<0.001) (**Figure 11**). Our model exhibits superior performance compared to the ISS staging system (**Figure 12**).

## 4 Discussion

Multiple myeloma is characterized by the uncontrolled proliferation of abnormal clonal plasma cells leading to destructive bone lesions, renal impairment, anemia, and hypercalcemia (29). Nowadays, patients with MM are expected to have increasing survival as a result of personalized treatments based on a better understanding of the biology and potential response to more effective therapies (30).These benefits are more pronounced in patients with high-risk diseases (31).Thus, it is critical to diagnosis and identify high-risk patients in order to move away from treatment adapted to patient’s physiological/chronological age and comorbidities and rather toward the establishment of risk-adapted treatment approaches. The conventional diagnosis and cytogenetic stratification of multiple myeloma relies on bone marrow aspiration and biopsy. Liquid biopsy offers a significant step forward compared with bone marrow aspiration because of its less invasive nature, lower cost, real-time insights into tumor status and, in some cases, the ability to overcome the issue of tumor heterogeneity (32).We selected a novel kind of liquid biopsy, namely circulating exosome miRNAs and constructed a diagnostic and prognostic model for MM, hoping to provide new strategies and methods for the diagnosis and early prognostic prediction of high-risk MM.

According to the miRNA sequencing results between NDMM and HC, we identified 20 miRNAs with significantly differential expression in MM and KEGG pathway enrichment analysis of the target genes for these differentially expressed miRNAs revealed that they predominantly clustered in the “MiRNAs in cancer”. In the testing stage, we observed a significant downregulation of miR-10a-5p, miR-10b-3p, miR-192-5p, miR-125a-5p, miR-483-3p, and let-7b-5p in MM. Besides, we developed a novel diagnostic model including six miRNAs which demonstrates outstanding performance with AUC 0.94, while also possessing high sensitivity (0.88) and high specificity (0.94). This strategy is expected to significantly improve the precision in the diagnosis of multiple myeloma. Some researchers have explored the diagnostic value of miRNAs in MM, for example, Kubiczkova observed a significant decrease in the expression of miR-744, miR-130a, let-7d, and let-7e in the MM group (33). However, most of them focused on the serum miRNAs not exosome-derived miRNAs, which were much more stable with protective membrane. Moreover, Zhang’s group found that most circulating plasma miRNAs are contained within exosomes and microvesicles, not in cell-free plasma (34). The protective membrane of extracellular vesicles stabilizes miRNAs, conferring higher credibility and stability to our selected biomarkers (35, 36).

Salomon performed a small RNA sequencing from circulating exosomes of 10 NDMMs and 5HCs. They identified 22 miRNAs, including let-7b, let-7c, let-7e, miR-10b, miR-125a, among others, whose expression levels in NDMMs were significantly reduced compared to HCs (27). They confirmed let-7b and miR-18a were significant predictors for OS in the multivariate models. We validated the aforementioned findings in 60 NDMMs and 20 HCs, confirming the down-expression of miR-10b-3p, miR-125a-5p, and let-7b-5p. Additionally, in a validation cohort of 130 patients, we demonstrated that reduced let-7b-5p expression correlates with poor prognosis, thus extending and confirming Salomon’s study findings in a larger population. However, the amplification of miR-18a was not successful, potentially due to variations in RNA provenance.

Zhang et al. identified 38 downregulated and 5 upregulated exosomal RNAs in MM compared to HC. Among these, let-7c-5p, let-7d-5p, miR-140-3p, miR-185-3p, and miR-425-5p were significantly reduced compared to the healthy control group. In the testing stage, it was found that the expression levels of let-7d-5p, miR-140-3p, and miR-425-5p in exosomes were significantly decreased compared to those of the healthy control group (37). Their findings partially align with our own research, as both studies observed a downregulation of the let-7 family in patients with multiple myeloma. The let-7 miRNA family members act as tumor suppressors. They negatively regulate the translation of oncogenes and cell cycle regulators including RAS, MYC and HMGA2. Let-7 miRNAs present decreased expression in several cancer types, and low let-7 levels correlate with poor prognosis (38). In contrast to the Zhang group’s use of microarray profiling for detecting differentially expressed genes, RNA-Seq is devoid of hybridization-based limitations such as background noise and saturation, and probe set issues including incorrect annotation and isoform coverage. RNA-Seq offers superior sensitivity for genes with low expression and accuracy for highly abundant genes (39). Furthermore, our study, with a testing cohort of 60 NDMMs and a validation cohort of 130 cases, provides more robust results compared to the smaller testing cohort of 48 NDMMs used by Zhang’s team.

We have identified that the plasma-circulating exosomal miR-483-3p, hsa-miR-125a-5p, and hsa-miR-10b-3p have significant diagnostic potential for multiple myeloma, with respective areas under the curve (AUCs) of 0.86, 0.77, and 0.75. Moreover, we selected three RNAs with significant differences, hsa-miR-193b-5p, miR-483-3p and let-7b-5p, to evaluate the prognostic value in 130 patients. The results showed that low expression of miR-483-3p and let-7b-5p is significantly associated with poor overall survival in MM. While hsa-miR-193b-5p is not related to the prognosis of patients. Our study highlighted both the diagnostic and prognostic value of miR-483-3p. It has been reported that the diagnostic performance of plasma miR-483-5p for MM is reflected by an area under the ROC curve of 0.745, demonstrating a sensitivity of 58% and a specificity of 90% (40). In concert with related studies, our results imply that the diagnostic potential of miRNAs derived from plasma and exosomes is comparable in the context of multiple myeloma diagnosis. Gu. et al. found that miR-483-5p was up-regulated in MM-MSCs (bone marrow-derived mesenchymal stem cells of MM patients) and could be transferred from MM-MSCs to MM cells via exosomes to favor MM progression by targeting TIMP2 (41). Interestingly, emerging evidence suggests that miR-483-5p may be involved in the pathogenesis of osteoporosis, and it has been proposed that the miR-483-5p/SATB2 axis could influence osteogenic differentiation and PI3K/AKT signaling (42). Based on these findings, it is plausible that miR-483-5p might also contribute to bone destruction, which is a common clinical manifestation of MM, though this remains speculative without direct functional evidence from the current study. Additionally, studies in clear cell renal cell carcinoma (ccRCC) have reported decreased miR-483-5p expression in tumor tissues compared to normal renal tissue, with elevated plasma levels observed post-nephrectomy (43, 44). In ccRCC, higher miR-483-5p expression was inversely correlated with inflammatory markers such as the neutrophil-to-lymphocyte ratio (NLR) and lymphocyte-to-monocyte ratio (LMR), and was shown to inhibit cancer cell proliferation and metastasis by modulating epithelial-mesenchymal transition (EMT) (43, 44). While these findings highlight a potential role for miR-483-5p in renal pathology and inflammation, its implications in renal impairment in MM patients remain hypothetical and require further investigation. Thus, although miR-483-5p may represent a candidate molecule linking bone and renal abnormalities in MM, these possibilities should be interpreted with caution and validated through dedicated mechanistic studies in future research.

Another important finding in our study is the diagnostic of exosome miR-10b. Hsa-miR-10b-3p studied have not been previously described in MM by literature. The deregulated expression level of hsa-miR-10b is a fundamental miRNA in various metastatic cancer (45). Based on the hub genes and their regulatory DEmiRs, researchers constructed miRNA-mRNA regulatory networks in MM that may be associated with the regulation of the bone microenvironment. These networks included hsa-miR-10b-5p-CDKN2A. Moreover, results revealed that the expression of the level of hsa-miR-10b is consistently deregulated in the MM cohorts. Suggests that the hsa-miR-10b-5p-CDKN2A networks may be contribute to the immunosuppression in MM (46, 47).

We further constructed a prognostic model of overall survival for MM based on the liquid biopsy methods. In our univariate Cox regression analysis, showed that aging, high ISS risk, high LDH, high creatinine and high bone marrow plasma cell ratio were adverse prognostic factors. We further integrated three miRNAs that significantly affect patient prognosis—hsa-miR-483-3p, let-7b-5p, and hsa-miR-193-5p—with clinical indicators to create a nomogram model capable of predicting 1-year, 3-year, and 5-year survival rates. Compared to the ISS staging system, our model effectively differentiated patients. Particularly for those in ISS stage III, where we provided further stratification. This model offers a novel method for risk stratification in multiple myeloma patients.

Although the Kaplan-Meier analysis of miR-193b-5p alone did not reach conventional statistical significance (p=0.052), we included it in our multivariate prognostic model based on its biological plausibility and complementary value. Previous studies have implicated miR-193b-5p in tumor progression in various cancers (48), and its variant miR-193b-3p has been identified as a key deregulated miRNA in myeloma cells, regulating MCL1 expression (28). Sensitivity analysis demonstrated that the model excluding miR-193b-5p did not outperform the original model (AUC=0.744), suggesting that it captures unique biological variance not fully accounted for by other miRNAs(**Supplementary File 2**). Its inclusion highlights a novel aspect of exosomal miRNA signatures in MM, warranting further mechanistic investigation.

Our study must also be considered in the context of pre-analytical variables, a well-established challenge in the reproducibility of liquid biopsy and exosome-based biomarker research (49). Factors such as sample collection tubes, processing delays, centrifugation protocols, and storage conditions can significantly impact exosome yield, purity, and molecular content. While our results demonstrate promising biomarker potential, we acknowledge that the generalizability of our findings may be influenced by our specific standardized protocols, as detailed in the Methods. We implemented stringent procedures—including defined centrifugation forces, strict processing time windows, batch-controlled exosome isolation, and quality control via NTA, TEM, and immunoblotting—to minimize this variability and ensure consistency across our sample set. Nonetheless, the absence of a universally standardized protocol across laboratories remains a significant hurdle for the clinical translation of exosomal miRNAs. Future multi-center validation studies, using harmonized using harmonized standard operating procedures, will be essential to confirm the robustness of our identified biomarkers and to definitively establish their clinical utility independent of pre-analytical influences.

However, our study has its limitations. Primarily, the sample size was small, and long-term follow-up is required to validate the relationship between the levels of hsa-miR-193b-5p, miR-483-3p, and let-7b-5p and patient prognosis, as well as the applicability of the new model. Furthermore, extensive and in-depth research is necessary to elucidate the mechanisms by which hsa-miR-193b-5p, miR-483-3p, and let-7b-5p contribute to the pathogenesis and progression of multiple myeloma.

In conclusion, liquid biopsies, facilitating the analysis of tumor-derived elements such as cells, nucleic acids (e.g., DNA, miRNA), and tumor-altered platelets within bodily fluids, present a superior diagnostic modality characterized by minimal invasiveness, enhanced replicability, and the capacity for both early-stage detection and continuous surveillance, effectively navigating the challenges posed by tumor heterogeneity compared to traditional tissue biopsies (50, 51). In MM, liquid biopsies serve as a valuable tool for the initial detection, longitudinal assessment of disease progression, therapeutic response evaluation (inclusive of post-relapse interventions), and risk stratification for metastatic potential, highlighting their profound clinical applicability (52–56). Our study leveraged the intrinsic stability and miRNA-enriched content of exosomes, employing next-generation RNA sequencing to delineate a panel of differentially expressed genes. Subsequently, we constructed a highly sensitive and specific diagnostic biomarker signature, alongside a prognostic model that amalgamates the expression profiles of three pivotal exosomal miRNAs. This novel biomarker ensemble is poised to enhance the stratification of survival outcomes at 1, 3, and 5 years, and its validation in extensive cohorts is anticipated to solidify its potential as a diagnostic and prognostic paradigm for multiple myeloma management.

## Data Availability

The original contributions presented in the study are publicly available. This data can be found here: https://www.ebi.ac.uk/ena/browser/home; PRJEB101067.
