# Supplementary material for: Identification and prognostic prediction of high-risk multiple myeloma by exosomal microRNA
Source: Front Oncol. 2025 Oct 31;15:1659708. doi: 10.3389/fonc.2025.1659708 (PMC12615177; doi:10.3389/fonc.2025.1659708)
Supplement: Supplementary file 1 [file DataSheet1.docx]

**Supplementary**

Supplementary Table 1 Missing values in the validation cohort are imputed using random interpolation.

| Variables | Validation stage | | *P* value |
| --- | --- | --- | --- |
|  | before- MI | after- MI |  |
| **Number** | 130 | 130 |  |
| **Gender** |  |  | 1 |
| Male | 80(61.5%) | 80(61.5%) |  |
| Female | 50(38.5%) | 50(38.5%) |  |
| **Age(median,range)** | 62(37,79) | 62(37,79) | 1 |
| **ISS** |  |  | 1 |
| I | 26(20%) | 26(20%) |  |
| II | 46(35.4%) | 46(35.4%) |  |
| III | 58(44.6%) | 58(44.6%) |  |
| **Lactate dehydrogenase (median, U/L)** | 162(133.5,203) | 164.5(134,204.5) | 0.835 |
| Missing | 4 |  |  |
| **Creatinine(mg/dl)** | 0.88(0.73,1.32) | 0.88(0.73,1.35) | 0.898 |
| Missing | 2 |  |  |
| **Serum Calcium(mmol/L)** | 2.23(2.06,2.38) | 2.25(2.09,2.38) | 0.677 |
| Missing | 30 |  |  |
| **White Blood Cell(10^9/L)** | 5.33(3.99,7.22) | 5.32(4.00,7.22) | 0.984 |
| Missing | 1 |  |  |
| **Hemoglobin(g/L)** | 93(73,114.5) | 93(73,114.25) | 0.985 |
| Missing | 1 |  |  |
| **Platelets(10^9/L)** | 157(113,198) | 156(113.5,198) | 0.976 |
| Missing | 1 |  |  |
| **Albumin(g/L)** | 35.8(29.83,40.05) | 35.8(29.78,39.95) | 0.930 |
| Missing | 2 |  |  |
| **TotalProtein(g/L)** | 84.8(71.2,106.6) | 84.75(70.95,106.7) | 0.962 |
| Missing | 7 |  |  |
| **Globulin(g/L)** | 48.3(30.08,73.4) | 48.3(30.23,73.85) | 0.971 |
| Missing | 6 |  |  |
| **Monoclonal protein(g/L)** | 35.15(13.88,48.33) | 33.95(13.03,48.03) | 0.591 |
| Missing | 18 |  |  |
| **Bone marrow plasma cell ratio(%)** | 33.5(16,52.5) | 33.75(16,51.75) | 0.948 |
| Missing | 3 |  |  |
| **Immunoparesis degree** |  |  | 0.999 |
| No | 11(8.5%) | 11(8.5%) |  |
| Mild | 11(8.5%) | 12(9.2%) |  |
| Moderate | 20(15.4%) | 21(16.2%) |  |
| Severe | 82(63.1%) | 86(66.2%) |  |
| Missing | 6 |  |  |
| **Heavy-chain type** |  |  |  |
| No | 36(27.7%) | 36(27.7%) | 1 |
| IgG | 66(50.8%) | 66(50.8%) |  |
| IgA | 28(21.5%) | 28(21.5%) |  |
| **Light-chain type** |  |  | 1 |
| kappa | 65(50%) | 65(50%) |  |
| lambda | 65(50%) | 65(50%) |  |
| **Alkaline phosphatase(U/L)** | 76(56,103) | 76(56.75,104.25) | 0.889 |
| Missing | 3 |  |  |

Supplementary Table 2 Sequences of primers and probes for the target gene and the internal reference gene

| Primer Name | Primer Sequence (5'--3'） |
| --- | --- |
| qPCR-TYR | GTGCAGGGTCCGAGGT |
| U6-RT | AACGCTTCACGAATTTGCGT |
| U6-S | CTCGCTTCGGCAGCACA |
| U6-A | AACGCTTCACGAATTTGCGT |
| U6 probe | AGAAGATTAGCATGGCCCCTGCGCA |
| hsa-miR-122-5p-RT | GTCGTATCCAGTGCAGGGTCCGAGGTATTCGCACTGGATACGACCAAACAC |
| hsa-miR-122-5p-F | CCGCTGGAGTGTGACAATG |
| hsa-miR-122-5p-P | TCGCACTGGATACGACCAAACAC |
| hsa-miR-192-5p-RT | GTCGTATCCAGTGCAGGGTCCGAGGTATTCGCACTGGATACGACGGCTGT |
| hsa-miR-192-5p-F | ACCGCCTGACCTATGAATTG |
| hsa-miR-192-5p-P | TCGCACTGGATACGACGGCTGT |
| hsa-miR-10a-5p-RT | GTCGTATCCAGTGCAGGGTCCGAGGTATTCGCACTGGATACGACCACAAA |
| hsa-miR-10a-5p-F | CGCTACCCTGTAGATCCGAA |
| hsa-miR-10a-5p-P | TTCGCACTGGATACGACCACAAATT |
| hsa-miR-10b-3p-RT | GTCGTATCCAGTGCAGGGTCCGAGGTATTCGCACTGGATACGACTATTCC |
| hsa-miR-10b-3p-F | ACCGCCCAAATTCGTATCT |
| hsa-miR-10b-3p-P | TTCGCACTGGATACGACTATTCCC |
| hsa-miR-125a-5p-RT | GTCGTATCCAGTGCAGGGTCCGAGGTATTCGCACTGGATACGACTCACAG |
| hsa-miR-125a-5p-F | CGTCCCTGAGACCCTTTAAC |
| hsa-miR-125a-5p-P | TCGCACTGGATACGACTCACAGGT |
| hsa-miR-148a-3p-RT | GTCGTATCCAGTGCAGGGTCCGAGGTATTCGCACTGGATACGACACAAAG |
| hsa-miR-148a-3p-F | ACCGTCAGTGCACTACAGA |
| hsa-miR-148a-3p-P | ATTCGCACTGGATACGACACAAAGT |
| hsa-miR-193b-5p-RT | GTCGTATCCAGTGCAGGGTCCGAGGTATTCGCACTGGATACGACTCATCT |
| hsa-miR-193b-5p-F | TAACGGGGTTTTGAGGGC |
| hsa-miR-193b-5p-P | ATTCGCACTGGATACGACTCATCTC |
| hsa-miR-483-3p-RT | GTCGTATCCAGTGCAGGGTCCGAGGTATTCGCACTGGATACGACAAGACG |
| hsa-miR-483-3p-F | CGCTCACTCCTCTCCTCC |
| hsa-miR-483-3p-P | TCGCACTGGATACGACAAGACGG |
| hsa-miR-9-5p-RT | GTCGTATCCAGTGCAGGGTCCGAGGTATTCGCACTGGATACGACTCATAC |
| hsa-miR-9-5p-F | AGGCGGTCTTTGGTTATCTAG |
| hsa-miR-9-5p-P | ATTCGCACTGGATACGACTCATACAG |
| hsa-miR-99b-5p-RT | GTCGTATCCAGTGCAGGGTCCGAGGTATTCGCACTGGATACGACCGCAA |
| hsa-miR-99b-5p-F | AATCACCCGTAGAACCGAC |
| hsa-miR-99b-5p-P | TTCGCACTGGATACGACCGCAAG |
| hsa-miR-200a-3p-RT | GTCGTATCCAGTGCAGGGTCCGAGGTATTCGCACTGGATACGACACATCG |
| hsa-miR-200a-3p-F | ACGCTAACACTGTCTGGTAAC |
| hsa-miR-200a-3p-P | TTCGCACTGGATACGACACATCG |
| hsa-miR-625-3p-RT | GTCGTATCCAGTGCAGGGTCCGAGGTATTCGCACTGGATACGACTGAGGG |
| hsa-miR-625-3p-F | GGCGGCGACTATAGAACTTT |
| hsa-miR-625-3p-P | TTCGCACTGGATACGACTGAGGG |
| let-7b-5p-RT | GTCGTATCCAGTGCAGGGTCCGAGGTATTCGCACTGGATACGACAACCAC |
| let-7b-5p-F | AGCCGCCTGAGGTAGTAG |
| let-7b-5p-P | TTCGCACTGGATACGACAACCAC |
| hsa-miR-18a-5p-RT | GTCGTATCCAGTGCAGGGTCCGAGGTATTCGCACTGGATACGACCTATCTG |
| hsa-miR-18a-5p-F | TCCGCTAAGGTGCATCTAGTG |
| hsa-miR-18a-5p-P | TCGCACTGGATACGACCTATCTGC |

Supplementary Table 3 Performance characteristics of miRNAs for diagnostic model in newly diagnosed multiple myeloma patients

| miRNA | Accuracy | NPV | Sensitivity | Specificity | AUC |
| --- | --- | --- | --- | --- | --- |
| hsa-miR-483-3p | 0.85 | 0.68 | 0.88 | 0.75 | 0.86 |
| hsa-miR-10a-5p | 0.79 | 0.60 | 0.9 | 0.45 | 0.78 |
| hsa-miR-125a-5p | 0.73 | 0.47 | 0.72 | 0.75 | 0.77 |
| hsa-miR-10b-3p | 0.70 | 0.45 | 0.62 | 0.95 | 0.75 |
| let-7b-5p | 0.60 | 0.38 | 0.48 | 0.95 | 0.68 |
| hsa-miR-192-5p | 0.59 | 0.36 | 0.50 | 0.85 | 0.66 |
| hsa-miR-99b-5p | 0.66 | 0.35 | 0.75 | 0.40 | 0.59 |
| hsa-miR-148a-3p | 0.56 | 0.33 | 0.50 | 0.75 | 0.57 |
| hsa-miR-122-5p | 0.58 | 0.33 | 0.55 | 0.65 | 0.56 |
| hsa-miR-193b-5p | 0.48 | 0.23 | 0.40 | 0.55 | 0.51 |

Abbreviations: NPV:Negative Predictive Value

| 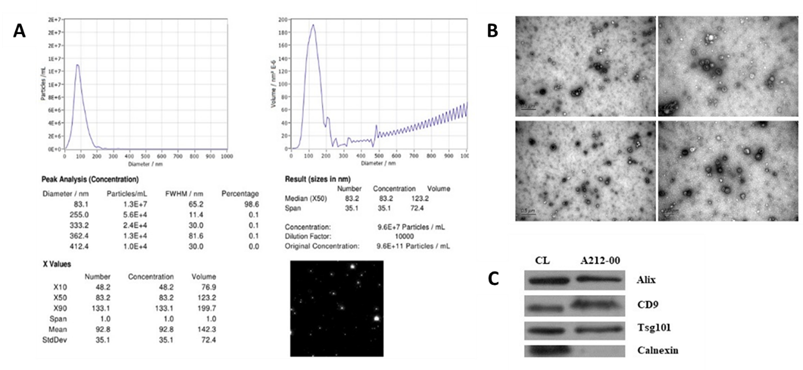 |
| --- |
| Figure S1. Identification of Circulating Exosomes in Multiple Myeloma:(A)Nanoparticle tracking analysis (NTA) identifies exosomes: The median size of exosomes that meet the definition is 83.2 nm;(B) Transmission electron microscopy identification of exosomes: Clear cup-shaped vesicular structures are visible in the microscope field, with a diameter consistent with the standards for exosomes;(C) Western Blot method for the identification of exosomes: The isolated exosomes specifically express Alix, CD9, and Tsg101, but do not express Calnexin. |
| 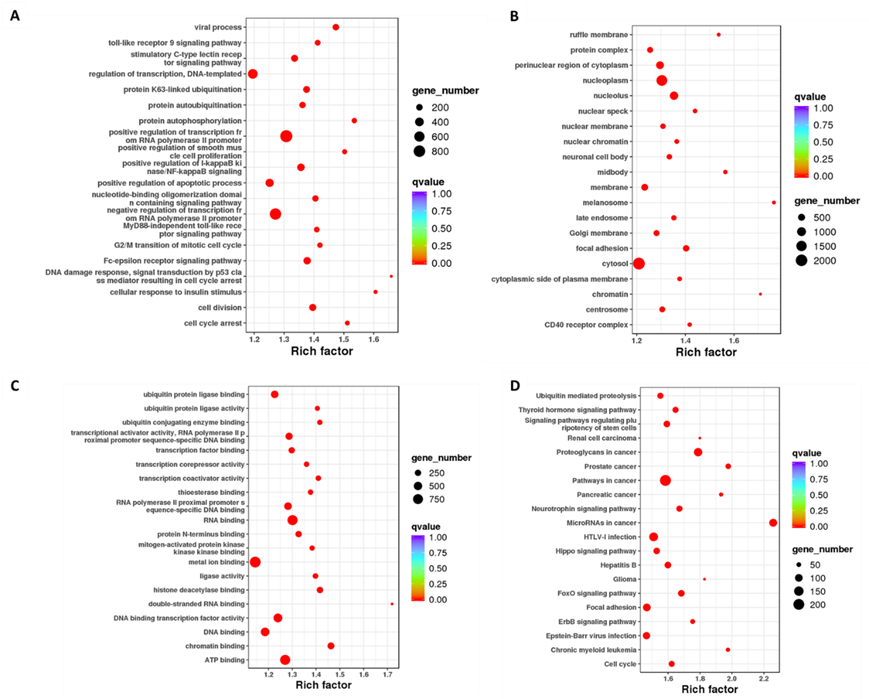 |
| Figure S2. Bubble chart of KEGG pathway enrichment for target genes of differentially expressed miRNAs-Target gene of up-regulation miRNA:(A)Significant bubble diagram of a biological process;(B)Significant bubble diagram of cell components;(C)Significant bubble diagram of a molecular function;(D)KEGG enrichment results |
| 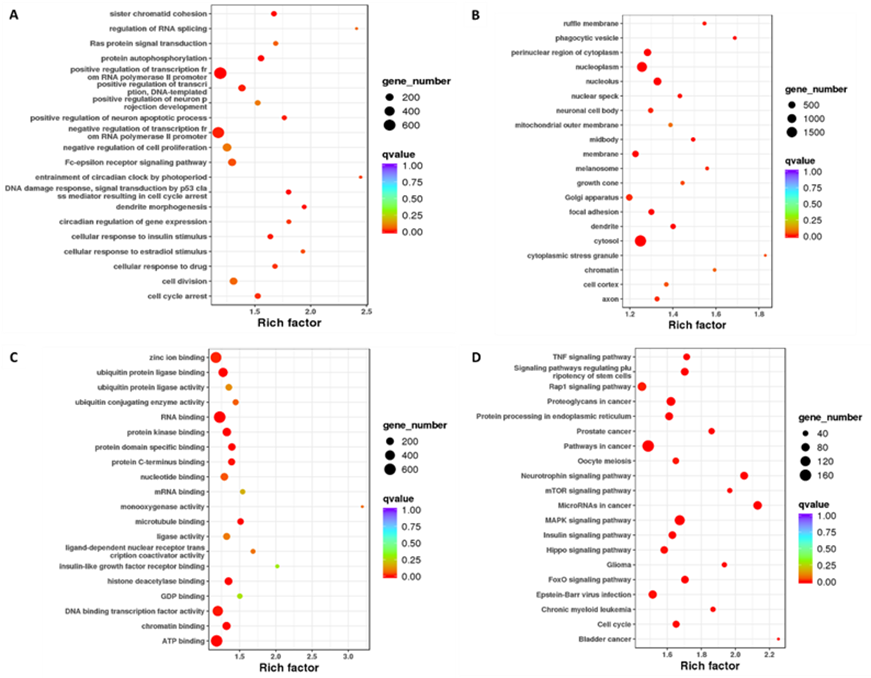 |
| Figure S3. Bubble chart of KEGG pathway enrichment for target genes of differentially expressed miRNAs-Target gene of down-regulation miRNA:(A)Significant bubble diagram of a biological process;(B)Significant bubble diagram of cell components;(C)Significant bubble diagram of a molecular function;(D)KEGG enrichment results |
