# Supplementary material for: Identification and prognostic prediction of high-risk multiple myeloma by exosomal microRNA
Source: Front Oncol. 2025 Oct 31;15:1659708. doi: 10.3389/fonc.2025.1659708 (PMC12615177; doi:10.3389/fonc.2025.1659708)
Supplement: Supplementary file 2 [file DataSheet2.docx]

Supplementary Table. Univariate and multivariable Cox regression analysis for overall survival of newly diagnosed multiple myeloma patients

|  | Univariate Cox regression analysis | | | multivariable Cox regression analysis | | | |
| --- | --- | --- | --- | --- | --- | --- | --- |
| Variables | HR | 95% CI | *P* value | | HR | 95%CI | *P* value |
| **Gender** |  |  | 0.12 | |  |  |  |
| Male | ref |  |  | |  |  |  |
| Female | 0.69 | (0.43,1.10) |  | |  |  |  |
| **Age** | 1.03 | (1.00,1.05) | 0.006 | | 1.03 | (1.01,1.06) | 0.006 |
| **ISS stage** |  |  | 0.005 | |  |  |  |
| I | ref |  |  | | ref |  |  |
| II | 1.53 | (0.76,3.10) | 0.236 | | 1.06 | (0.5,2.21) | 0.883 |
| III | 2.59 | (1.34,5.03) | 0.005 | | 1.34 | (0.63,2.84) | 0.447 |
| **Lactate dehydrogenase** | 1.01 | (1.00,1.01) | 0.005 | | 1.15 | (0.92,1.44) | 0.208 |
| **Creatinine** | 1.13 | (1.04,1.23) | 0.004 | | 1.08 | (0.98,1.20) | 0.133 |
| **Serum Calcium** | 1.49 | (0.84,2.64) | 0.177 | |  |  |  |
| **Hemoglobin** | 0.99 | (0.98,1.00) | 0.072 | |  |  |  |
| **Monoclonal protein** | 0.99 | (0.98,1.00) | 0.156 | |  |  |  |
| **Bone marrow plasma cell ratio** | 1.01 | (1.00,1.02) | 0.007 | | 1.01 | (1.00,1.02) | 0.022 |
| **Heavy-chain type** |  |  | 0.09 | |  |  |  |
| No | ref |  |  | |  |  |  |
| IgG | 0.60 | (0.36,1.00) | 0.051 | |  |  |  |
| IgA | 0.94 | (0.52,1.70) | 0.843 | |  |  |  |
| **Light-chain type** |  |  |  | |  |  |  |
| kappa | ref |  |  | |  |  |  |
| lambda | 1.31 | (0.84,2.02) | 0.232 | |  |  |  |
| **Immunoparesis degree** |  |  | 0.1 | |  |  |  |
| No | ref |  |  | |  |  |  |
| Mild | 1.44 | (0.50,4.14) | 0.503 | |  |  |  |
| Moderate | 0.65 | (0.23,1.88) | 0.431 | |  |  |  |
| Severe | 1.47 | (0.63,3.40) | 0.369 | |  |  |  |
| **miRNA score** |  |  | 0.01 | |  |  |  |
| 0 | ref |  |  | | ref |  |  |
| 1 | 1.16 | (0.57,2.36) | 0.684 | | 1.05 | (0.50,2.18) | 0.902 |
| 2 | 2.06 | (1.26,3.36) | 0.004 | | 1.92 | (1.15,3.20) | 0.01 |

| 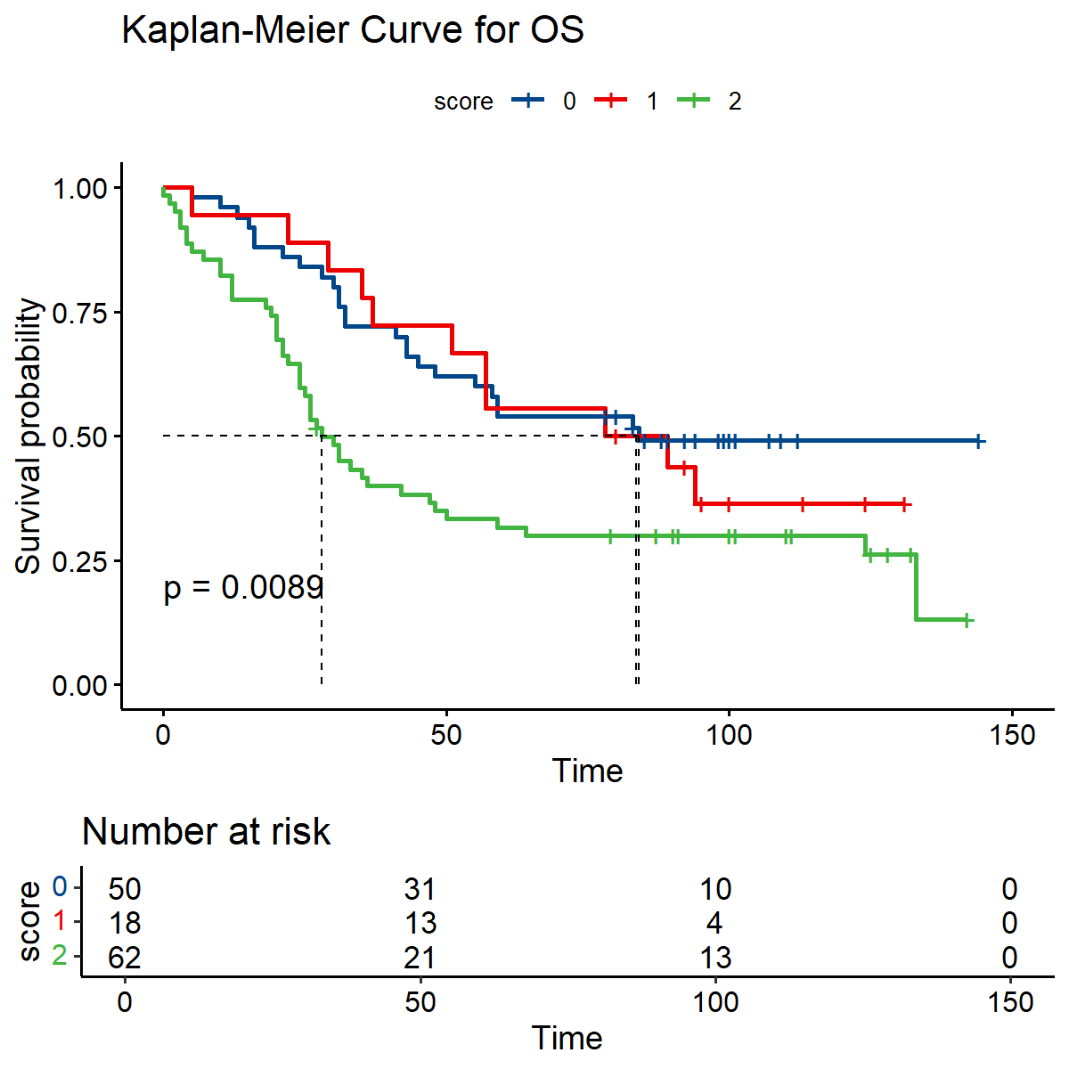 |
| --- |
| K-M curves were plotted based on different scores in a validation set of 130 newly diagnosed multiple myeloma, with each low expression of the two prognostic biomarkers assigned a score of 1. |
| 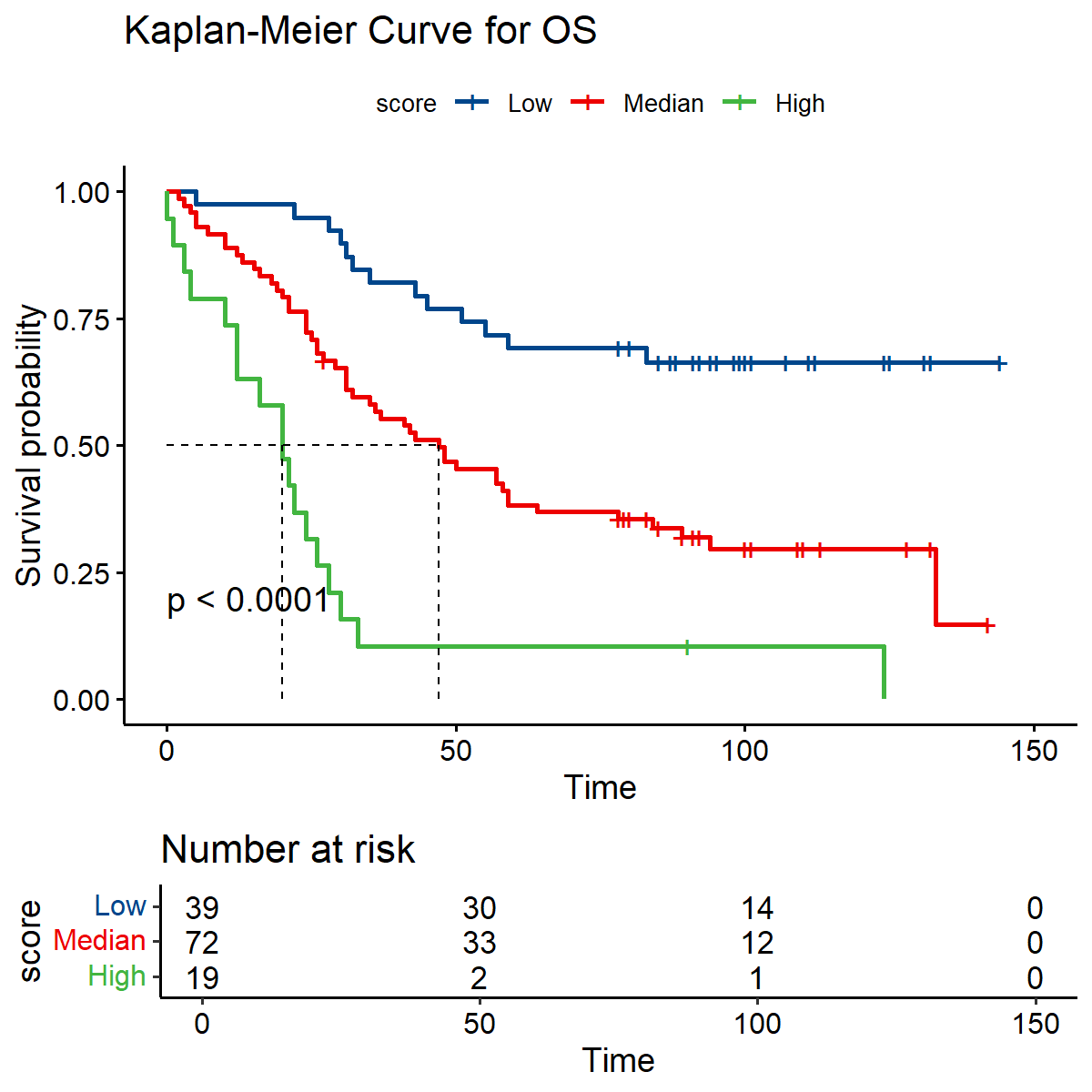 |
| K-M curve shows the difference in OS due to the norm- stage. |
| 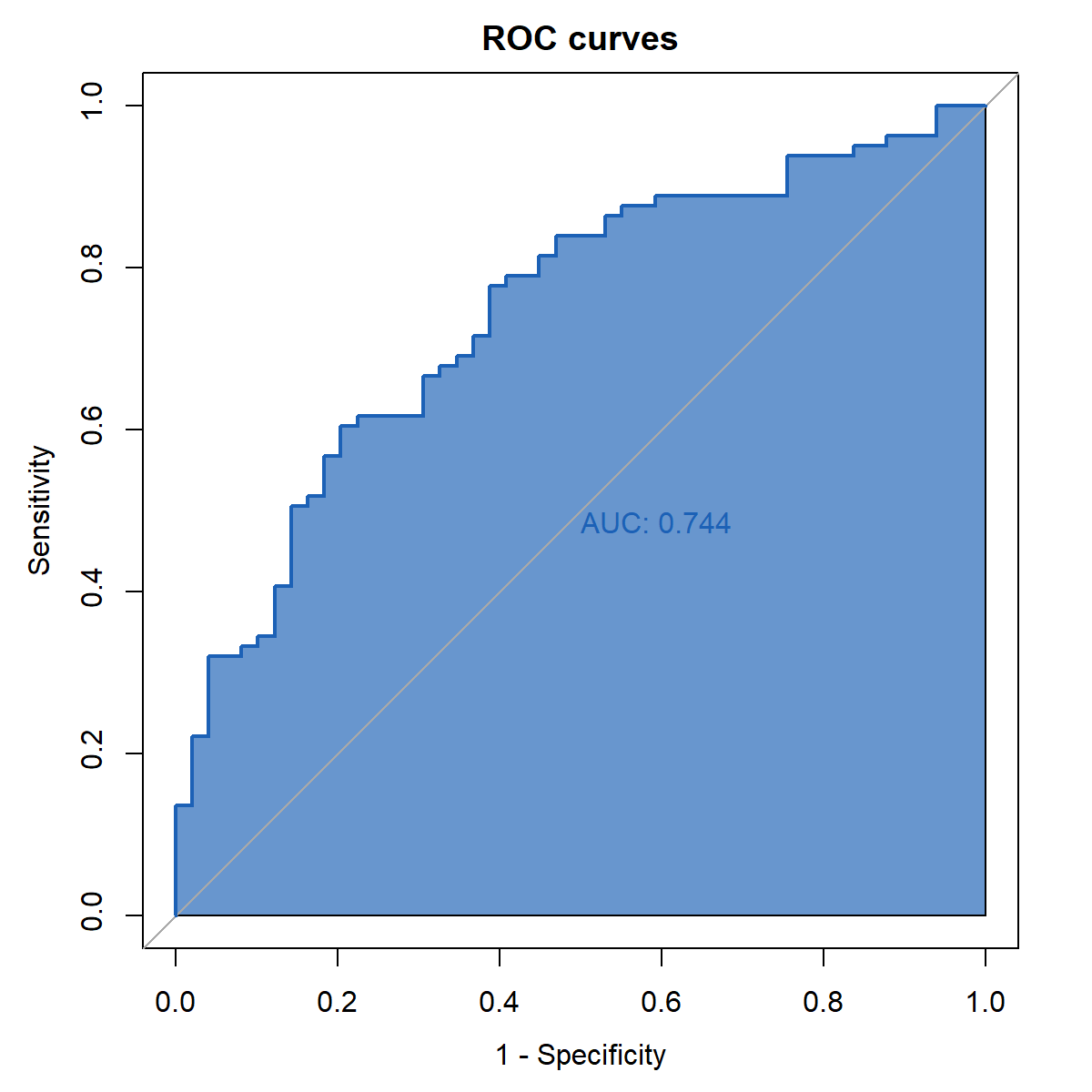 |
| AUC curve of the norm- stage for predicting OS; |
